# Corrigendum: Quercetin suppresses ovariectomy-induced osteoporosis in rat mandibles by regulating autophagy and the NLRP3 pathway

**DOI:** 10.3389/ebm.2024.10149

**Published:** 2024-05-17

**Authors:** Yue Xiong, Cheng-Wei Huang, Chao Shi, Liang Peng, Yu-Ting Cheng, Wei Hong, Jian Liao

**Affiliations:** ^1^ Department of Prosthodontics and Implantology, School/Hospital of Stomatology, Guizhou Medical University, Guiyang, China; ^2^ AOSI CAR Dental, Shantou, China; ^3^ Guizhou Medical University, Guiyang, China

**Keywords:** NLRP3, Quercetin, alendronate, autophagy, osteoclasts, postmenopausal osteoporosis

In the published article, there was a mistake in Figure 6 and Figure 7 as published. In the original published version, the authors incorrectly reversed the labeling of the positions of LC3I and LC3II when labeling the names of the proteins. The corrected Figure 6 and Figure 7 appears below.

**FIGURE 6:**
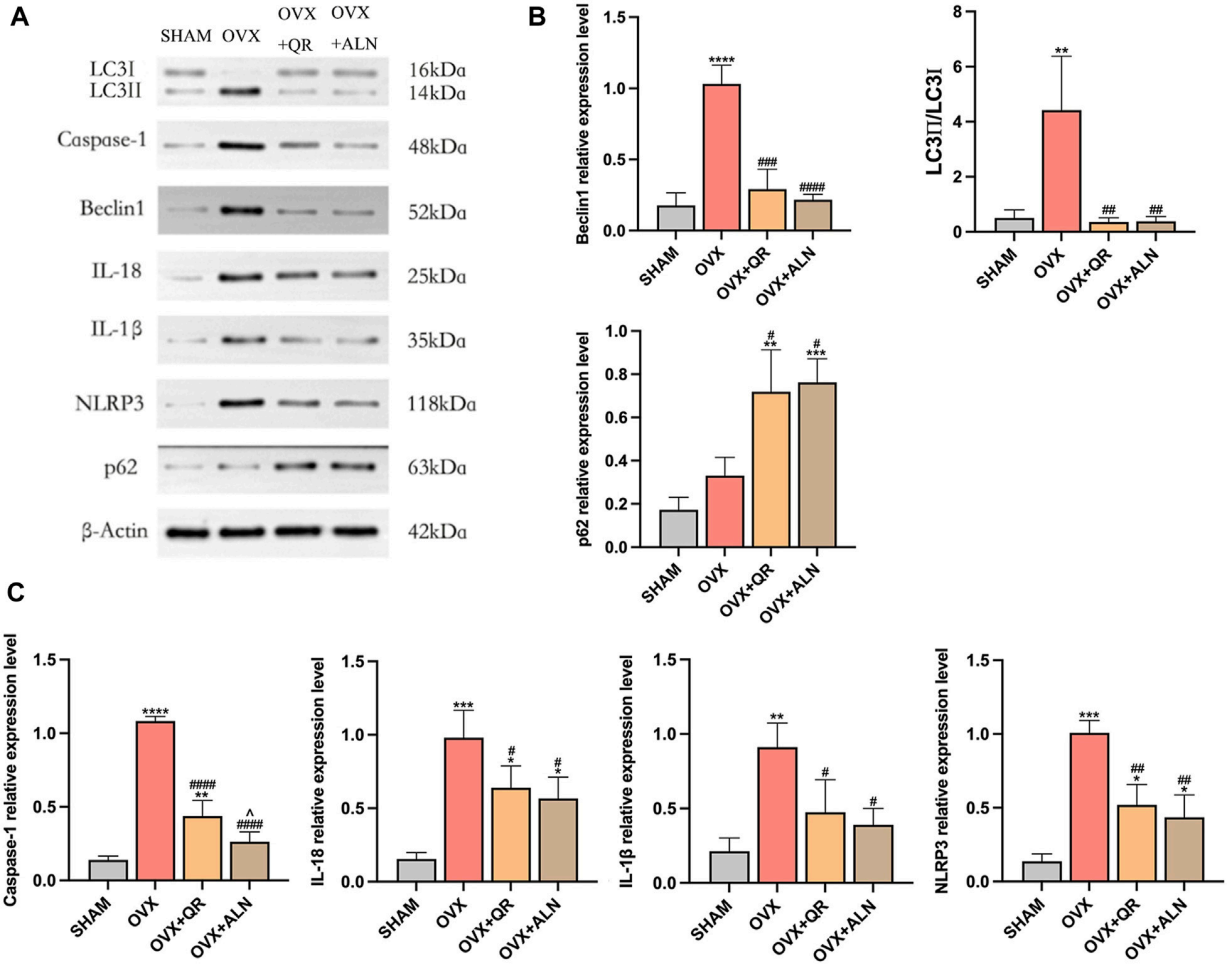
Effect of OVX and treatment using QR or ALN on the expression of NLRP3 pathway-related proteins as well as autophagy-related proteins. **(A)** Grayscale values of protein strips; **(B)** Western blotting showed the expression of Beclin1, p62, and LC3II/I ratio; and **(C)** Western blotting showed the expression of caspase-1, IL-18, IL-1β, and NLRP3. Data are expressed as mean (SD) of *n* = 6 from three independent experiments. *p < 0.05, **p < 0.01, ***p < 0.001, ****p < 0.0001 compared with SHAM group.^#^ *p* < 0.05, ^##^ *p* < 0.01, ^###^ *p* < 0.001, ^####^ *p* < 0.0001 compared with OVX group. ^^^ *p* < 0.05 compared with OVX + QR group.

**FIGURE 7:**
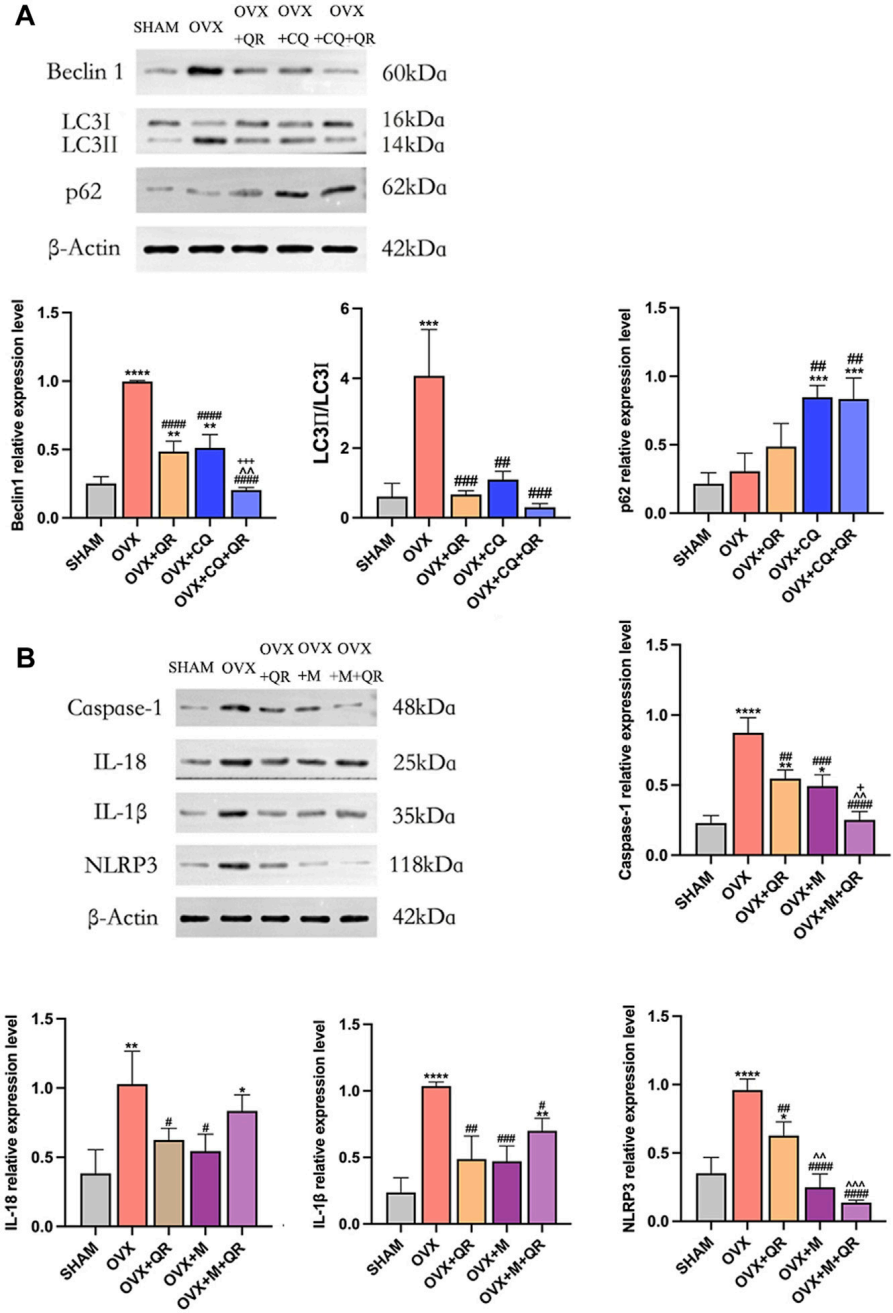
**(A)** Western blotting showed the effect of QR and CQ on the expression of autophagy-related proteins and **(B)** Western blotting showed the effect of QR and MCC950 on the expression of NLRP3 pathway-related proteins. Data are expressed as mean (SD) of *n* = 6 from three independent experiments. *p < 0.05, **p < 0.01, ***p < 0.001, ****p < 0.0001 compared with SHAM group. ^#^ *p* < 0.05, ^##^ *p* < 0.01, ^###^ *p* < 0.001, ^####^ *p* < 0.0001 compared with OVX group. ^^^^ *p* < 0.01, ^^^^^ *p* < 0.001 compared with OVX + QR group. ^+^ *p* < 0.05, ^+++^ *p* < 0.001 compared with OVX + CQ group or OVX + M group.

The authors apologize for this error and state that this does not change the scientific conclusions of the article in any way.

